# Yak-Kong Soybean (*Glycine max*) Fermented by a Novel *Pediococcus pentosaceus* Inhibits the Oxidative Stress-Induced Monocyte–Endothelial Cell Adhesion

**DOI:** 10.3390/nu11061380

**Published:** 2019-06-19

**Authors:** Ji Seung Kim, Jong Hun Kim, Sasikumar Arunachalam Palaniyandi, Charles C. Lee, Ji Woo You, Hee Yang, Jung Han Yoon Park, Seung Hwan Yang, Ki Won Lee

**Affiliations:** 1 Major in Biomodulation, Department of Agricultural Biotechnology, Seoul National University, Seoul 08826, Korea; jjuj07@naver.com (J.S.K.); gpwls3892@naver.com (J.W.Y.); 2 Research Institute of Agriculture and Life Sciences, Seoul National University, Seoul 08826, Korea; jonghunkim@sungshin.ac.kr (J.H.K.); yhee6106@snu.ac.kr (H.Y.); jyoon@hallym.ac.kr (J.H.Y.P.); 3 Department of Food Science and Biotechnology, Sungshin University, Seoul 01133, Korea; 4 Department of Biotechnology, Mepco Schlenk Engineering College, Mepco Engineering College, Sivakasi, Tamilnadu 626005, India; apsasikumar@mju.ac.kr; 5 Department of Food Science, Cornell University, Ithaca, NY 14853, USA; cclee4@gmail.com; 6 Advanced Institutes of Convergence Technology, Seoul National University, Suwon 16229, Korea; 7 Department of Biotechnology, Chonnam National University, Yeosu, Chonnam 59626, Korea

**Keywords:** Yak-Kong, small black soybean, *Pediococcus pentosaceus*, atherosclerosis prevention, monocyte–endothelial adhesion

## Abstract

Yak-Kong (YK), a small black soybean (*Glycine max*) in Korea, contained higher concentrations of antioxidants than ordinary black soybean or yellow soybean in our previous study. We prepared the fermented YK extract by using a novel lactic acid bacterium, *Pediococcus pentosaceus* AOA2017 (AOA2017) isolated from *Eleusine coracana*, and found that the antioxidant ability was enhanced after fermentation. In order to investigate the cause of the enhanced antioxidant ability in the fermented YK extract, we conducted a phenolic composition analysis. The results show that proanthocyanidin decreased and phenolic acids increased with a statistical significance after fermentation. Among the phenolic acids, p-coumaric acid was newly produced at about 11.7 mg/100 g, which did not exist before the fermentation. Further, the fermented YK extract with increased p-coumaric acid significantly inhibited the lipopolysaccharide-induced THP-1 monocyte–endothelial cell adhesion compared to the unfermented YK extract. The fermented YK extract also suppressed the protein expression levels of vascular cell adhesion molecule (VCAM)-1 in human umbilical vein endothelial cells (HUVECs). Together with the previous studies, our results suggest that the extract of YK fermented by AOA2017 has potential to be a new functional food material with its enhanced bioactive compounds which may help to prevent atherosclerosis caused by oxidative stress.

## 1. Introduction

Cardiovascular diseases (CVDs) are one of the greatest risk factors of human death globally. They are mostly associated with the development of atherosclerosis, which is a chronic inflammatory disease often induced by oxidative stress [1,2,3]. In the early stage of atherosclerosis, oxidative stress in endothelial cells can induce the expression of cellular adhesion molecules such as vascular cell adhesion molecule 1 (VCAM-1), which helps monocytes in the blood bind to endothelial cells [4]. Various sources, including bacterial lipopolysaccharides (LPS), inflammatory cytokines, and oxidized lipids, can stimulate oxidative stress in endothelial cells and increase reactive oxygen species (ROS). Therefore, the antioxidant activity can be used as an indicator for selecting and developing a substance having a preventive effect on CVDs.

Soybean (*Glycine max*) and its products, including fermented soybean sauces, are an integral part of the diet in eastern Asia. Many constituents of soybean, such as protein, soyasaponins, isoflavones, proanthocyanidins, and polysaccharides, are well known for their health-promoting effects [5,6,7,8,9,10]. Especially, soybean protein is known to reduce the incidence of coronary heart disease (CHD), and soybean phenolic compounds including isoflavones and proanthocyanidins have been well studied for their potential to prevent chronic diseases by their antioxidant and anti-inflammatory effects [6,8,9,10,11,12,13,14]. In Korea, there is a cultivar of soybean with a black seed coat and green embryo that is commonly called Yak-Kong (YK). It has a long history of being used as a medicinal herb, and recent studies have reported its antioxidant and anti-inflammatory effects [12,13,14]. In our previous study, we investigated the phenolic composition of YK soybean (*Glycine max*) and found that YK has a stronger antioxidant activity compared to common black and yellow soybeans. We also found that the YK embryo (rich in isoflavones) and YK seed coat (rich in proanthocyanidin) both strongly attenuated the adhesion of monocytes to endothelial cells [8].

Although fermentation is a traditional method for producing functional foods, it is still a useful method for developing valuable bioactive compounds in the pharmaceutical, nutraceutical, and food industries. It has been reported that soymilk fermentation with lactic acid bacteria leads to the formation of phenolic acids with enhanced bioactivity, antioxidant activity, and tumor suppression [15,16,17]. However, there have been few studies on the development of functional food materials using fermented soymilk for the prevention of CVDs. Before this study, we sought to identify lactic acid bacteria that are effective for the fermentation–bioconversion of YK to enhance the preventive effect on CVDs. Since it is known that oxidative stress plays an important role in the development of CVDs, we hypothesized that if the metabolites of YK produced or increased by lactic acid bacteria fermentation increased the antioxidant activity, it would enhance the preventive activity of YK on the adhesion of monocytes to endothelial cells. In this study, fermented YK extract was prepared using our novel *Pediococcus pentosaceus* AOA2017 (AOA2017), which was one of the most effective lactic acid bacteria for YK fermentation, and we investigated the preventive effect of fermented YK extract on LPS-induced monocyte–endothelial cell adhesion and VCAM-1 expression, which mimics the early stage of atherosclerosis.

## 2. Materials and Methods

### 2.1. Chemicals and Reagents

Yak-Kong soybean (*Glycine max*) was obtained from Daehak soymilk (Pyeongchang, Korea) and p-coumaric acid was from Chemfaces (Wuhan, Hubei, China, ≥98.9%). Calcein acetoxymethyl (AM) dye, fetal bovine serum (FBS), hydrocortisone, medium199 (M199), 2-mercaptoethanol, and lipopolysaccharides (LPS) were purchased from Sigma-Aldrich (St. Louis, MO, USA). RPMI 1640 medium was purchased from Welgene (Daegu, Korea). L-glutamine, fetal bovine serum (FBS), basic fibroblast growth factor (bFGF), and recombinant human epidermal growth factor (hEGF) were obtained from Gibco (Grand Island, NY, USA). Penicillin (10,000 units/mL)–streptomycin (10,000 µg/mL) (P/S) was from Corning (Corning, NY, USA). Antibodies of vascular cell adhesion molecule-1 (VCAM-1) and β-actin were purchased from Santa Cruz Biotechnology Inc. (Santa Cruz, CA, USA).

### 2.2. Microorganism and Bean Extract Fermentation

The strain *Pediococcus pentosaceus* AOA2017 was obtained from *Eleusine coracana* and used as a starter culture for the fermentation of YK soybean. Cultures were activated twice in deMan, Rogosa, and Sharpe (MRS) media (BD Biosciences, San Jose, CA, USA) and incubated at 37 °C for 24 h prior to use.

Whole YK was washed and soaked in distilled water overnight. Then, 200 g of YK was ground with 1 L of distilled water (ratio 1:5, *w*/*v*) and filtered with cheesecloth, and insoluble residues were separated. The slurry was then autoclaved at 121 °C for 15 min. After cooling, it was poured into the sterile tubes, and the inoculum was added at a 4% (*v*/*v*) rate. On completion of inoculation, the tubes were kept in an incubator at 37 °C. Samples were aseptically taken at 0 and 24 h and used as unfermented YK extract and fermented YK extract, respectively. Non-inoculated YK extract was treated with the same experimental conditions and taken as control for further analysis.

### 2.3. Analysis of Antioxidant Activity

#### 2.3.1. DPPH Free Radical Scavenging Activity

The 1,1-diphenyl-2-picrylhydrazyl (DPPH) radical scavenging activity of the sample was evaluated by the method of Blosis [18]. Briefly, 0.5 mL of DPPH alcoholic solution (0.2 mM) was mixed with 1 mL of sample and incubated in a test plate at 37 °C for 10 min. The absorbance of the mixture was measured at 517 nm in an automated microplate reader (Tecan Group Ltd., Männedorf, Switzerland). A blank was prepared without adding extract.

The inhibition percentage of the DPPH absorbance was calculated by the following:

DPPH scavenging % = (1 − (Abs of sample/Abs of blank)) × 100.

#### 2.3.2. ABTS Free Radical Scavenging Activity

ABTS (2,2′-azinobis-(3-ethylbenzothiazoline-6-sulphonic acid)) radical cation scavenging assay was determined by the method of ABTS cation decolorization assay [19]. First, 7 mM ABTS solution was mixed with 2.4 mM potassium persulfate and incubated for 16 h at room temperature in the dark. The mixture was then diluted by mixing 1 mL ABTS^.+^ solution with distilled water to obtain an absorbance of 0.70 ± 0.02 units. Sample extracts (10 μL) were allowed to react with 190 μL of the ABTS solution for 3 min in a dark condition. Then, the absorbance was measured at 734 nm using a UV/visible spectrophotometer. A blank was prepared without adding extract.

The scavenging activity was derived as follows:

= (1 − (Abs of sample /Abs of blank)) × 100.

#### 2.3.3. Reducing Power Assay

The reducing power assay was performed by the method of Yen [20]. The methanolic extract was mixed with 0.2 M phosphate buffer (pH 6.6) and 1% potassium ferricyanide (*w*/*v*). The mixture was incubated at 50 °C for 20 min. Afterward, 10% trichloroacetate was added, and the mixture was centrifuged at 3000 g for 10 min. The supernatant was collected, and Milli-Q water was added to it. Finally, 1% ferric chloride was mixed, and the absorbance of the mixture was measured at 700 nm. Absorbance intensity served as the measurement of the antioxidant activity of the extract.

### 2.4. Analysis of Phenolic Composition

#### 2.4.1. UHPLC–MS/MS Analysis

Phenolic acids were analyzed using UHPLC–MS/MS system (Waters Ltd., Milford, MA, USA) by the method of Dudonné et al. [21] with the same parameters used in the proanthocyanidin analysis. All phenolic compounds were quantified by gallic acid equivalents (GAE) and the data were acquired in negative ionization mode through multiple reaction monitoring (MRM).

#### 2.4.2. UHPLC–PDA Analysis

Isoflavones were analyzed using reversed-phase UHPLC with a Waters Acquity UPLC coupled to a PDA detector (Milford, MA, USA). The compounds were separated at 30 °C on a Waters Acquity HSS Cyano column (2.1 mm × 50 mm, 1.8 μm). The mobile phase consisted of solvent A with 0.1% formic acid in water and solvent B with 0.1% formic acid in acetonitrile mixed using the following gradient: 0–0.36 min, 10% B; 0.36–3.6 min, 10–30% B; 3.6–3.96 min, 30% B; 3.96–4 min, 10% B; 4–6 min, 10% B. The flow rate was 0.58 mL/min, and the injection volume was 1 μL. Chromatographic data were acquired at 260 nm. A calibration curve of a daidzein standard was used for quantification of daidzein, glycitein, and their derivatives, while genistein was quantified using its corresponding standards.

#### 2.4.3. HPLC-Fluorescence Analysis

Proanthocyanidins were analyzed using normal-phase analytical HPLC with fluorescence by the method of Dudonné et al. [21]. The fluorescence was measured at 230 nm of excitation and 321 nm of emission wavelengths. Proanthocyanidins were separated by degrees of polymerization (DP) from 1 to >10. They were quantified by an external calibration curve of epicatechin, applying a correction factor according to their respective response in fluorescence.

### 2.5. Cell Culture

Human umbilical vein endothelial cells (HUVECs) were obtained from Lonza (Walkersville, MD, USA) and cultured in M199 with 25 mM 4-(2-hydroxyethyl)-1-piperazineethanesulfonic acid (HEPES) containing three growth factors (1 ng/mL hydrocortisone, 1 ng/mL hEGF, and 2 ng/mL bFGF), 1% (*v*/*v*) penicillin-streptomycin, 2 mM L-glutamine, and 10% (*v*/*v*) FBS (Gibco). Cells were used between passages 7 and 14. The human monocyte-like leukemia cell line THP-1 was purchased from the Korean Cell Line Bank and grown in RPMI 1640 medium containing 50 µM 2-mercaptoethanol, 1% (*v*/*v*) penicillin-streptomycin, and 10% (*v*/*v*) FBS (Sigma-Aldrich). Cells were incubated at 37 °C in 5% CO_2_ and the density of THP-1 was maintained between 2 × 10^5^ and 1 × 10^6^ cells/mL.

### 2.6. THP-1 Monocyte Adhesion Assay

Confluent HUVECs in 96-well plates were pretreated with fermented and unfermented YK extract with AOA2017 and p-coumaric acid for 1 h and stimulated with 100 ng/mL LPS for 5 h. THP-1 cells labeled with calcein AM were added to the HUVECs at a density of 5 × 10^5^ cells/well in M199. After 1 h incubation, the non-adhered THP-1 cells were washed with phosphate-buffered saline (PBS). Florescence was measured with an Infinite 200 PRO system (Tecan Group Ltd., Männedorf, Switzerland) at excitation and emission wavelengths of 485 nm and 538 nm, respectively.

### 2.7. Western Blot Assay

Confluent HUVECs in 6-well plates were treated with fermented and unfermented YK extract with AOA2017 and p-coumaric acid for 1 h. Pre-treated cells were stimulated with 100 ng/mL LPS for 5 h. The cells were washed with cold PBS and scraped with lysis buffer to obtain cell lysates. The protein lysates with equal amounts were separated by 10% sodium dodecyl sulfate–polyacrylamide gel electrophoresis and transferred onto polyvinylidene difluoride membranes. The membranes were blocked with 5% skim milk and incubated with specific primary antibodies (1:1000) at 4 °C overnight. After incubating the membranes with horseradish peroxidase-conjugated secondary antibodies (1:10,000) for 1 h, protein bands were visualized using an enhanced chemiluminescence detection kit (GE Healthcare, London, UK).

### 2.8. Measurement of Intracellular ROS

HUVECs were seeded in black 96-well plates and pretreated with p-coumaric acid for 1 h and stimulated with LPS (100 ng/mL) for 3 h. Then, 96-well plates were loaded with 20 μM of 2′,7′-dichlorofluorescin diacetate (DCF-DA, Sigma-Aldrich, St. Louis, MO, USA) for 20 min and washed with phosphate-buffered saline (PBS). The fluorescence was determined by using an Infinite 200 PRO system (Tecan Group Ltd., Männedorf, Switzerland) at excitation and emission wavelengths of 485 nm and 538 nm, respectively.

### 2.9. Statistical Analysis

Statistical analyses were performed using SPSS (Statistical Analysis System Institute 2010, IBM corp., Chicago, IL, USA). Data are expressed as the mean ± standard deviation of the mean (SD). When only two groups were compared, the student’s *t*-test was used. For more than two groups, data were analyzed using the one-way analysis of variance (ANOVA) followed by Tukey’s honest significant difference test. Statistical significance was set at *p* < 0.05.

## 3. Results

### 3.1. Fermentation Using P. pentosaceus AOA2017 Increases the Antioxidant Activity of YK

**Figure 1:**
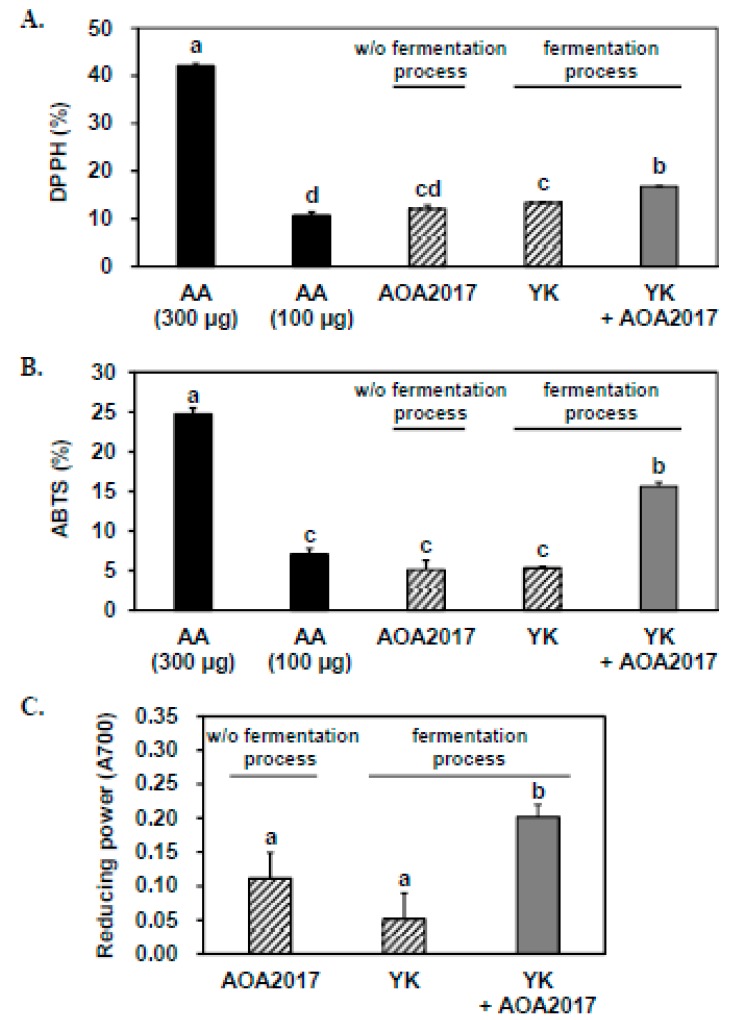
Effect of fermentation using *Pediococcus pentosaceus* AOA2017 on the antioxidant activity of Yak-Kong (YK). The antioxidant activity was compared among three samples: the bacterial broth (*P. pentosaceus* AOA2017) without the fermentation process, the extract of YK applied with the fermentation process without *P. pentosaceus* AOA2017, and the extract of YK fermented by *P. pentosaceus* AOA2017. (**A**) Antioxidant activity measured by 1,1-diphenyl-2-picrylhydrazyl (DPPH) free radicals, (**B**) Antioxidant activity measured by 2,2′-azinobis-(3-ethylbenzothiazoline-6-sulphonic acid, ABTS) free radicals, and (**C**) the reducing power. Data are expressed as mean ± SD (*n* = 3). Data were analyzed using one-way analysis of variance (ANOVA) followed by Tukey’s honest significant difference test. Different letters (a, b, c, and d) on the top of the bars indicate significant differences (*p* < 0.05) among different treatments. AA, ascorbic acid; YK, Yak-Kong; AOA2017, *P. pentosaceus* AOA2017; w/o, without.

Antioxidant activity was used to select lactic acid bacteria suitable for YK fermentation among our library of lactic acid bacteria. To determine the effect of YK fermentation using AOA2017 on antioxidant activity, we performed DPPH, ABTS, and reducing power assays with the fermented YK extract. In order to exclude the effect of lactic acid bacteria itself and the effect of chemical changes of YK during the fermentation process without lactic acid bacteria, we used appropriate samples for negative controls as shown in Figure 1. When comparing the scavenging effects on DPPH and ABTS free radicals with the same amount of the three sample extracts, both negative controls showed similar antioxidant activity as 100 μg of ascorbic acid, and the fermented YK extract showed significantly higher antioxidant activity than the two negative controls (Figure 1A,B). The antioxidant activity of the fermented YK extract was approximately two to four times higher than the negative controls with the reducing power assay (Figure 1C). These results indicate that the changes made by the fermentation using AOA2017 increases the antioxidant activity of YK.

### 3.2. Fermentation of YK Using P. pentosaceus AOA2017 Increases the Content of Phenolic Acids and Decreases Proanthocyanidins

**Figure 2:**
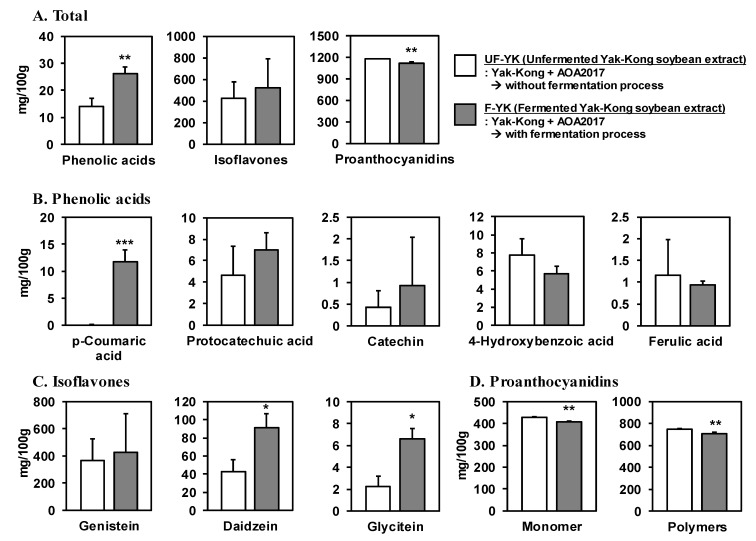
Effect of fermentation using *P. pentosaceus* AOA2017 on the content of polyphenols in YK. (**A**) Total content of phenolic acids, isoflavones, and proanthocyanidins in unfermented and fermented YK extract. The content of the major compounds were measured using (**B**) UHPLC-MS/MS, (**C**) UHPLC-PDA, and (**D**) HPLC-fluorescence analysis, respectively. Data are expressed as mean ± SD (*n* = 3). Student’s *t*-test was used for statistical analysis between UF-YK and F-YK; *** *p* < 0.001, ** *p* < 0.01, * *p* < 0.05. UF-YK, unfermented Yak-Kong extract; F-YK, Yak-Kong extract fermented with *P. pentosaceus* AOA2017.

Since polyphenols play an important role in antioxidant activity, we conducted a comprehensive analysis of the phenolics in the fermented YK extracts to investigate the effects of AOA2017 fermentation. For comparison, we used the microbial culture containing a mixture of YK and AOA2017 before fermentation. Fermented YK extract contained 26.24 ± 2.39 mg phenolic acids per 100 g, an increased value that was two times higher than the unfermented YK extract (*p* < 0.01), while there was a significant decrease in the proanthocyanidin content (1178.2 ± 3.96 mg/100 g to 1115.1 ± 17.21 mg/100 g, *p* < 0.01) after fermentation. However, there was no significant change in the content of total isoflavones (Figure 2A).

Among the 51 kinds of phenolics in the analysis library, only five phenolic acids—including p-coumaric acid, protocatechuic acid, catechin, 4-hydroxybenzoic acid, and ferulic acid—were measured by UHPLC–MS/MS. p-Coumaric acid was newly generated after the fermentation for which the content in the fermented YK extract was 11.7 ± 2.21 mg/100 g (*p* < 0.001). However, no significant changes were observed in the other phenolic acids (Figure 2B). Further, isoflavones were measured by UHPLC–PDA, and daidzein (90.9 ± 16.08 mg/100 g) and glycitein (6.6 ± 0.98 mg/100 g) were significantly increased about two and three times in the fermented YK extract, respectively, compared to the unfermented YK extract (*p* < 0.05) (Figure 2C). Proanthocyanidins of various degrees of polymerization (DP) were analyzed by HPLC with fluorescence. The contents of the proanthocyanidin monomers (406.2 ± 3.44 mg/100 g) and polymers (DP ≥10,708.9 ± 13.80 mg/100 g) in the fermented YK extract were significantly decreased compared to the unfermented YK extract (*p* < 0.01) (Figure 2D).

### 3.3. Fermented YK Extract and Its Bioactive Compound p-Coumaric Acid Have a Strong Inhibitory Effect on LPS-Stimulated THP-1 Monocyte–Endothelial Cell Adhesion

**Figure 3:**
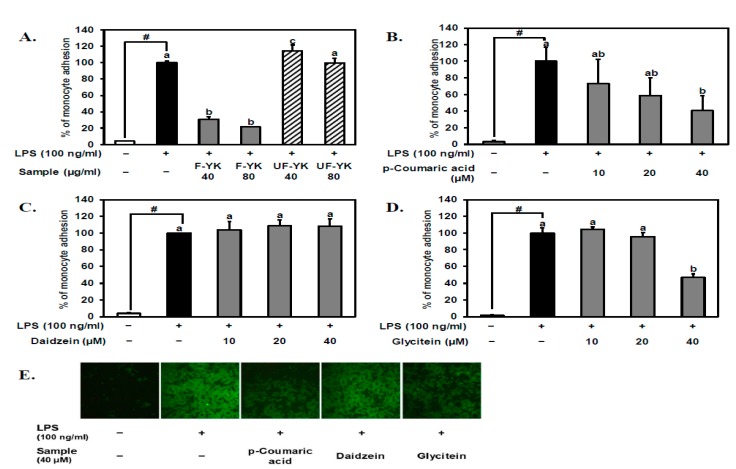
Effect of fermented YK extract and its bioactive compound p-coumaric acid on lipopolysaccharide (LPS)-stimulated THP-1 monocyte–endothelial cell adhesion. After pretreatment with (**A**) unfermented, fermented YK extract, (**B**) p-coumaric acid, (**C**) daidzein, and (**D**) glycitein at various concentrations for 1 h, human umbilical vein endothelial cells (HUVECs) were induced by LPS for 5 h. (**E**) Representative images of THP-1 monocyte–endothelial cell adhesion with 40 μM of p-coumaric acid, daidzein, and glycitein. Calcein AM-labeled THP-1 monocytes were added to HUVECs for 1 h and washed with phosphate-buffered saline (PBS). Quantification of adhered THP-1 cells was conducted as described in the Materials and Methods. Data from A to D are expressed as mean ± SD (*n* = 3). Student’s *t*-test was used for statistical analysis between control and LPS treatment; # (*p* < 0.05). Data were analyzed using one-way analysis of variance (ANOVA) followed by Tukey’s honest significant difference test. Different letters (a, b, and c) on the top of the bars indicate significant differences (*p* < 0.05) among sample treatments with LPS. UF-YK, unfermented Yak-Kong extract; F-YK, Yak-Kong extract fermented with *P. pentosaceus* AOA2017; +, treated with LPS or samples, −, not treated with LPS or samples.

The preventive effect of the fermented YK extract on the early stage of atherosclerosis was evaluated *in vitro* using the monocyte adhesion assay. 1-h pretreatments on HUVECs with 40 and 80 μg/mL of the fermented YK extract strongly inhibited the interaction between THP-1 monocytes and LPS-induced HUVECs compared to the unfermented YK extract with the same concentrations (Figure 3A). p-Coumaric acid, which was the specific phenolic acid newly produced during the fermentation of YK, also suppressed the adhesion between THP-1 monocytes and LPS-induced HUVECs in a dose-dependent manner as shown in Figure 3B. Other isoflavones, daidzein, and glycitein, which significantly increased after YK fermentation, were also tested. Daidzein did not show any effect under 40 μM (Figure 3C), but the least-existing isoflavone glycitein significantly inhibited the LPS-induced THP-1 monocyte–endothelial cell adhesion only at 40 μM (Figure 3D). Image comparison of the fluorescent monocytes attached to the endothelial cells clearly shows that the molecules that exist in a small portion may contribute to the preventive effect of the fermented YK extract (Figure 3E). These results indicate that the fermented YK extract has the potential to prevent the LPS-induced early stage of atherosclerosis, at least in part, due to the generation of p-coumaric acid and/or the increase in glycitein. Considering the amount existing in the fermented YK extract and the tendency of dose-dependent inhibition on the monocyte–endothelial cell adhesion, p-coumaric acid could be the most important bioactive polyphenol to contribute to the enhanced preventive effect of the fermented YK extract.

### 3.4. Fermented YK Extract and Its Bioactive Compound p-Coumaric Acid Decrease VCAM-1 Expression Levels in LPS-Stimulated Endothelial Cells

**Figure 4:**
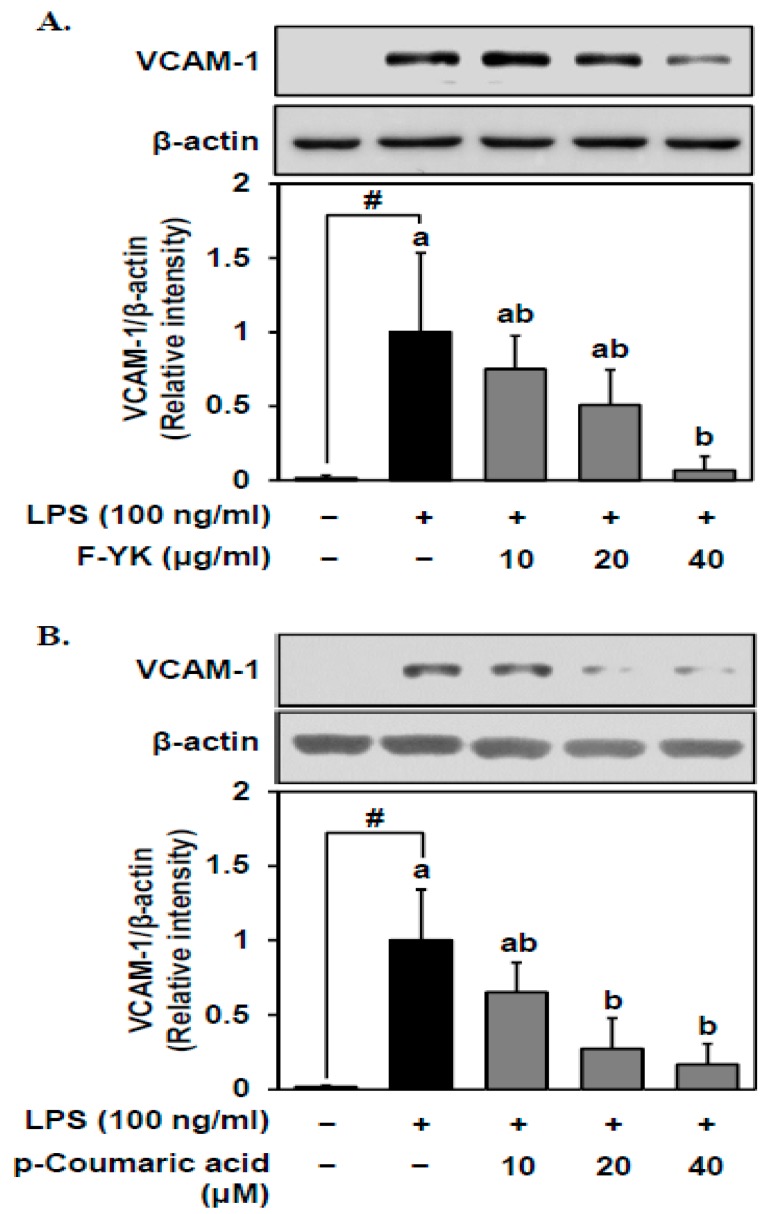
Effect of fermented YK extract and its bioactive compound p-coumaric acid on vascular cell adhesion molecule (VCAM)-1 expression levels in LPS-stimulated endothelial cells. After pretreatment with (**A**) fermented YK extract and (**B**) p-coumaric acid of various concentrations for 1 h, HUVECs were stimulated with LPS for 5 h. VCAM-1 protein expression was measured by Western blot analysis as described in the Materials and Methods. Data are expressed as mean ± SD (*n* = 3). Student’s *t*-test was used for statistical analysis between control and LPS treatment; # (*p* < 0.05). Data were analyzed using one-way analysis of variance (ANOVA) followed by Tukey’s honest significant difference test. Different letters (a and b) on the top of the bars indicate significant differences (*p* < 0.05) among different sample treatments with LPS. F-YK, Yak-Kong extract fermented with *P. pentosaceus* AOA2017; +, treated with LPS or samples, −, not treated with LPS or samples.

To assess the effects of the fermented YK extract on the LPS-induced expression of vascular cell adhesion molecule (VCAM)-1, Western blot analysis was conducted. VCAM-1 protein expression levels decreased in the LPS-induced HUVECs by 10–40 μg/mL of the fermented YK extract in a dose-dependent manner (Figure 4A). Similarly, p-coumaric acid, a potent bioactive compound of the fermented YK extract, downregulated the VCAM-1 protein expression in a dose-dependent manner (Figure 4B).

### 3.5. p-Coumaric Acid, the Bioactive Compound of Fermented YK Extract, Decreases the LPS-Stimulated ROS Production in Endothelial Cells

**Figure 5:**
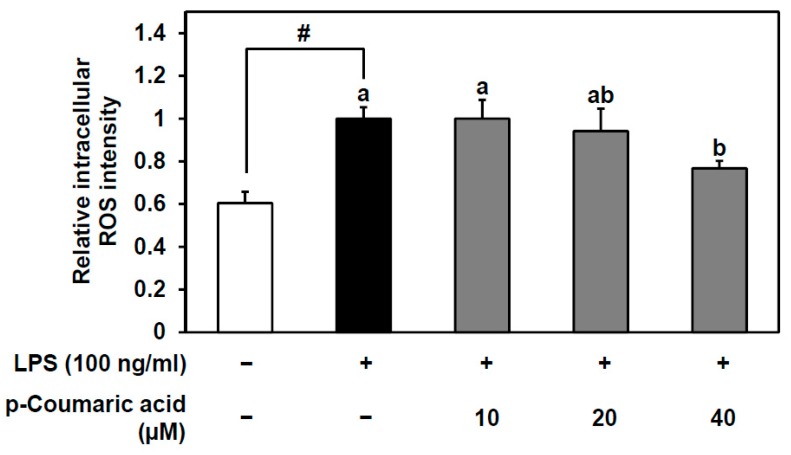
Antioxidant effect of p-coumaric acid on LPS-induced intracellular reactive oxygen species (ROS). After pretreatment with 10, 20, and 40 μM p-coumaric acid for 1 h, HUVECs were stimulated with LPS for 3 h. Relative intracellular ROS was measured using the cell-permeable fluorogenic probe 2′,7′-dichlorofluorescin diacetate (DCF-DA) as described in the Materials and Methods. Data are expressed as mean ± SD (*n* = 3). Student’s *t*-test was used for statistical analysis between control and LPS treatment; # (*p* < 0.05). Data were analyzed using one-way analysis of variance (ANOVA) followed by Tukey’s honest significant difference test. Different letters (a and b) indicate significant differences (*p* < 0.05) among samples treatments with LPS; +, treated with LPS or p-coumaric acid, −, not treated with LPS or p-coumaric acid.

To confirm the antioxidant effect of p-coumaric acid inside HUVECs, the DCF-DA assay was conducted. Intracellular ROS levels were significantly increased with 3 h of LPS treatment. Pretreating the cells with 40 μM of p-coumaric acid significantly inhibited the LPS-induced increase in intracellular ROS (Figure 5). Since p-coumaric acid is known as an antioxidant polyphenol, this result shows that the enhanced antioxidant capacity of the fermented YK is at least partly due to p-coumaric acid both outside and inside the cells. As we showed that the fermented YK extract and its bioactive compound p-coumaric acid inhibits the LPS-induced VCAM-1 expression and monocyte–endothelial cell adhesion (Figure 3 and Figure 4), it can be predicted that these effects involve redox signaling and p-coumaric acid can play a role in enhancing the bioactivity of fermented YK.

## 4. Discussion

AOA2017 is a novel lactic acid bacterium that we discovered for the first time. While we were screening the most effective lactic acid bacteria for YK fermentation from our lactic acid bacteria library, the top priority was to consider whether the lactic acid bacteria grow well in the culture medium containing YK and whether the antioxidant capacity of the YK component was enhanced. It is widely known that phenolic acids have scavenging power on ROS [22,23]. According to a previous study, microbial action can metabolize proanthocyanidin and produce phenolic acids with low molecular weight. Human colonic microflora catabolized proanthocyanidin polymers into non-hydroxylated aromatic acids and phenolic acids with a hydroxyphenyl group. Within 48 h of incubation, most of the proanthocyanidin polymers were nearly degraded [24]. In another previous study, proanthocyanidin in sorghum was degraded to 52% and phenolic acid was produced during alcoholic fermentation with lactic acid bacteria [25]. Our results are consistent with both of these studies (Figure 2A).

The most interesting compound that significantly increased during the YK fermentation with AOA2017 was p-coumaric acid (Figure 2B). Since it was newly produced from 0 to 11.7 ± 2.21 mg/100 g, it is considered to be the most specific product of YK fermentation with the AOA2017 strain. p-Coumaric acid is a kind of hydroxycinnamic acid, which exists widely in many foods, such as apples, bananas, potatoes, and cabbages [26]. It is known that p-coumaric acid can be produced by the metabolism of microorganisms [27]. Recent studies indicated that p-coumaric acid has various bioactivities on antioxidant, anti-inflammatory, anti-obesity, and neuroprotective effects [28,29,30,31,32]. Further, it was shown that 10 µM of p-coumaric acid inhibited the LPS-induced monocyte adhesion and also suppressed VCAM-1 protein expression in endothelial cells [33], which was confirmed in our study (Figure 3B and Figure 4B). Other compounds that significantly increased during YK fermentation with AOA2017 were daidzein and glycitein (Figure 2C). It has been reported that soybean isoflavones show a protective effect on atherosclerosis by inhibiting various pathogenic mechanisms [34], and genistein and daidzein can inhibit monocyte–endothelial cell adhesion [35]. So far, the effect of glycitein (6.6 ± 0.98 mg/100 g after AOA2017 fermentation) on monocyte–endothelial cell adhesion has been unknown. As shown in Figure 3D,E, glycitein may also have the potential to inhibit the LPS-induced monocyte–endothelial cell adhesion. Therefore, our results suggest that the enhanced inhibitory effect of AOA2017-fermented YK extract on monocyte–endothelial cell adhesion may be partially attributed to the increased production of p-coumaric acid, daidzein, and glycitein.

The enhanced antioxidant capacity of fermented YK extract can be closely related to the preventive effect on CVDs. This is because excessive oxidative stress induces unstable homeostasis, endothelial dysfunction, and lipid peroxidation, which lead to the development of CVDs, including atherosclerosis, hypertension, and hyperlipemia [36,37,38,39]. Previous publications have shown that LPS induces the production of intracellular ROS [4,40,41]. There are various exogenous sources and endogenous cellular sources of ROS, including nicotinamide adenine dinucleotide phosphate (NADPH) oxidases, cellular organs such as mitochondria, peroxisomes, and endoplasmic reticulum, and auto-oxidation. In addition, reactive nitrogen species (RNS) which are mainly produced by nitric oxide synthases also play an important role in cellular oxidative stress. Many cellular functions are regulated by various concentrations of ROS or RNS. However, it is the excessive oxidative stress that causes trouble. Therefore, the endothelium has an important role in maintaining homeostasis from excessive ROS accumulation. It expresses antioxidant enzymes such as superoxide dismutase, catalase, and glutathione peroxidases (GPx) to prevent endothelial dysfunction. A recent study suggested that GPx-1 regulates the expression of adhesion molecules and proinflammatory cytokines, which contribute to monocyte–endothelial cell adhesion [42]. When LPS-stimulated endothelial cells were treated with antioxidants, the expression of VCAM-1 and intracellular adhesion molecule-1 (ICAM-1) was decreased [4]. There is cumulative evidence that cellular antioxidant effects in the blood vessels can prevent CVDs, but it is very difficult to understand the full mechanism due to its complexity. Cellular antioxidant effects can be acquired by both decreasing oxidative stress and increasing the activity of endogenous antioxidant enzymes. The DCF-DA assay, which we used in this study, is one of the widely used methods for detecting intracellular hydrogen peroxide and oxidative stress [43]. Further studies are necessary to investigate the precise mechanism of the cellular antioxidant effect of fermented YK.

We demonstrated the anti-atherosclerosis effect of fermented YK by the degree of inhibition of the LPS-induced THP-1 monocyte–endothelial cell adhesion *in vitro* assay, which mimics the condition of the early stage of atherosclerosis. In the early phase of atherosclerosis, LPS, inflammatory cytokines, or oxidized LDL promotes the protein expression of adhesion molecules such as VCAM-1 and ICAM-1 and the secretion of monocyte chemoattractant protein 1 (MCP-1) in endothelial cells. Further, LPS stimulation of endothelial cells can also induce the production of inflammatory cytokines, including TNF-α and IL-8, which can cause cardiovascular complications [41]. These responses of endothelial cells are often regulated by NF-kB signaling [9,44]. Then, leucocytes, such as THP-1 monocytes that flow in the blood vessels, attach to and transmigrate through the vascular endothelium by the adhesion molecules, and further aggravate the condition of atherosclerosis [1]. We previously showed that YK seed coat and embryo both strongly attenuated the LPS-induced THP-1 monocyte–endothelial cell adhesion in this same model [8]. However, this is the first time showing the enhanced preventive effect of the fermented YK extract on LPS-induced THP-1 monocyte–endothelial cell adhesion.

It is possible to think about the advantages of using lactic acid bacteria fermentation for bioconversion. First, it can be easily applied to foods for making and using enhanced physiologically active substances. Second, along with the probiotic function of lactic acid bacteria itself, we can expect the bioconversion of active substances inside our body. Although our results need to be verified in precise *in vivo* experiments before applying it to functional food development, our findings show that the extract of YK fermented by AOA2017 has a potential to be a new functional food material with its enhanced bioactive compounds which may help to prevent atherosclerosis caused by oxidative stress.

## 5. Conclusions

YK has a strong antioxidant activity due to its content rich of proanthocyanidin and isoflavones, and strongly attenuated the adhesion of monocytes to endothelial cells. Fermentation of YK by a novel lactic acid bacterium, *Pediococcus pentosaceus* AOA2017 (AOA2017) significantly enhanced the antioxidant ability and the inhibition of LPS (oxidative stress)-induced THP-1 monocyte–endothelial cell adhesion mainly due to the increase of p-coumaric acid. Therefore, this application of fermented YK can provide us a novel functional food material and a novel bioconversion method, which may help us to prevent CVDs caused by oxidative stress.
